# Association between enhanced carbonyl stress and decreased apparent axonal density in schizophrenia by multimodal white matter imaging

**DOI:** 10.1038/s41598-023-39379-w

**Published:** 2023-07-27

**Authors:** Shuraku Son, Makoto Arai, Kazuya Toriumi, Christina Andica, Daisuke Matsuyoshi, Koji Kamagata, Shigeki Aoki, Takahiko Kawashima, Takanori Kochiyama, Tomohisa Okada, Yasutaka Fushimi, Yuji Nakamoto, Yuko Kobayashi, Toshiya Murai, Masanari Itokawa, Jun Miyata

**Affiliations:** 1 Department of Psychiatry, Graduate School of Medicine, Kyoto University, 54 Shogoin-Kawaharacho, Sakyo-Ku, Kyoto, 606-8507 Japan; 2 Project for Schizophrenia Research, Tokyo Metropolitan Institute of Medical Science, Tokyo, Japan; 3 Department of Radiology, Juntendo University Graduate School of Medicine, Tokyo, Japan; 4 Institute of Quantum Life Science, National Institutes for Quantum Science and Technology, Takasaki, Japan; 5 Araya, Inc., Tokyo, Japan; 6 Brain Activity Imaging Center, ATR-Promotions, Kyoto, Japan; 7 Human Brain Research Center, Graduate School of Medicine, Kyoto University, Kyoto, Japan; 8 Department of Diagnostic Imaging and Nuclear Medicine, Graduate School of Medicine, Kyoto University, Kyoto, Japan

**Keywords:** Schizophrenia, Translational research, Magnetic resonance imaging, Endocrine system and metabolic diseases

## Abstract

Carbonyl stress is a condition featuring increased rich reactive carbonyl compounds, which facilitate the formation of advanced glycation end products including pentosidine. We previously reported the relationship between enhanced carbonyl stress and disrupted white matter integrity in schizophrenia, although which microstructural component is disrupted remained unclear. In this study, 32 patients with schizophrenia (SCZ) and 45 age- and gender-matched healthy volunteers (HC) were recruited. We obtained blood samples for carbonyl stress markers (plasma pentosidine and serum pyridoxal) and multi-modal magnetic resonance imaging measures of white matter microstructures including apparent axonal density (intra-cellular volume fraction (ICVF)) and orientation (orientation dispersion index (ODI)), and inflammation (free water (FW)). In SCZ, the plasma pentosidine level was significantly increased. Group comparison revealed that mean white matter values were decreased for ICVF, and increased for FW. We found a significant negative correlation between the plasma pentosidine level and mean ICVF values in SCZ, and a significant negative correlation between the serum pyridoxal level and mean ODI value in HC, regardless of age. Our results suggest an association between enhanced carbonyl stress and axonal abnormality in SCZ.

## Introduction

Carbonyl stress is a state caused by increased rich reactive carbonyl compounds (RCOs)^1^, which facilitate the formation of advanced glycation end products (AGEs), including pentosidine. AGEs have been associated with not only various age-related illnesses, such as cardiovascular events^2^, heart failure^3^, and Alzheimer’s-type dementia^4^, but also schizophrenia^5^. Enhanced carbonyl stress, reflected on high plasma pentosidine and low serum pyridoxal (a measurable vitamin B6 in equilibrium with pyridoxamine, a scavenger one) levels, has been reported in about 20% of patients with schizophrenia^5^. For this sub-population, a novel treatment using pyridoxamine, a kind of vitamin B6, is expected to reduce carbonyl stress effectively^6^.

Diffusion tensor imaging (DTI) is a magnetic resonance imaging (MRI) method commonly used for examining human white matter, and white matter alteration in schizophrenia has been frequently reported^7–12^. The underlying microstructural pathology is unclear, together with inconsistent postmortem findings^8,13,14^. However, we previously reported that enhanced carbonyl stress was associated with reduced white matter fractional anisotropy (FA, a general measure of white matter integrity) in schizophrenia^15^.

The latest in-vitro studies reported that glyoxalase 1 (GLO1), an essential enzyme for detoxifying RCOs, -knockout iPS cells exhibited impaired neurospheres and shortened neurites (= axon + dendrite)^16^ related to mitochondrial dysfunction^17^. Additionally, one postmortem study revealed that a schizophrenia case with GLO1 frameshift gene mutation showed curved and frizzy neurites^18^. Another in-vitro study demonstrated that cultivation of dorsal root ganglion cells with reduced pyridoxal levels decreased myelination and increased AGE levels^19^. Yet another postmortem study revealed AGE deposits in the cytoplasm of neurons as well as decreased myelin density in a schizophrenia patient’s brain^20^. Thus, it is still unclear whether axon or myelin is associated with carbonyl stress.

DTI assumes a widely used one-compartment ellipsoid model for water diffusion in MRI voxels, but it has not been sufficiently established to be equivalent to white matter microstructural histopathology. Free water imaging (FWI) models water diffusion by two compartments of tissue (intra- and extra-neurite) and CSF/edema spaces^21^, with the latter having been used as a surrogate marker of inflammatory change^22^. The Neurite Orientation Dispersion and Density Imaging (NODDI)^23^ models three compartments—intra-neurite (neurite = dendrite and axon), extra-neurite (space defined by membranes of somas and glial cells), and cerebrospinal fluid (CSF/edema) compartments. Combining these multiple modalities of different characteristics enables the microstructural breakdown of the white matter histopathology of schizophrenia.

In this study, we aimed to investigate which white matter microstructure is associated with enhanced carbonyl stress in schizophrenia using advanced multimodal MRI measures. We hypothesized that enhanced carbonyl stress in schizophrenia was related to the disruption of axons mediated by inflammatory change as was also assumed in our previous DTI study^15^.

## Results

### Demographics and clinical data

Details of the demographic and clinical data are shown in Table 1. Age and gender did not differ between groups.

**Table 1:** Demographic and clinical data in healthy and patient groups.

	Schizophrenia	Control	Effect size (*r*, Cramer’s *V*)	*P* value
N	32	45		
Age	43.0 [21]	40.00 [11]	− 0.124	0.277^a^
Gender (male/female)	20/12	21/24	0.156	0.170^b^
Pentosidine (µg/ml)	0.049 [0.022]	0.037 [0.014]	− 0.496	< 0.001^a^*
Pyridoxal (ng/ml)	6.90 [6.18]	9.00 [6.95]	− 0.136	0.232^a^
Age at onset	22.00 [13]	–		–
Duration of illness (years)	18.00 [16]	–		–
CP equivalent^c^ (mg/day)	550.00 [503.75]	–		–
PANSS positive	13.50 [8]	–		–
PANSS negative	19.50 [8]	–		–
PANSS general	30.00 [17]	–		–
tSNR	23.30 [3.28]	23.24 [2.31]		0.836^a^

All data are shown as median values [IQR].

*PANSS* Positive and Negative Syndrome Scale, *tSNR* temporal signal-to-noise ratio.

^a^Mann-Whitney U test.

^b^χ^2^.

^c^chlorpromazine (CP) equivalent was calculated according to the Practice Guidelines for the Treatment of Schizophrenia Patients.

*P < 0.001.

### Group differences of carbonyl stress markers (Table 1, Fig. 1A)

**Figure 1:**
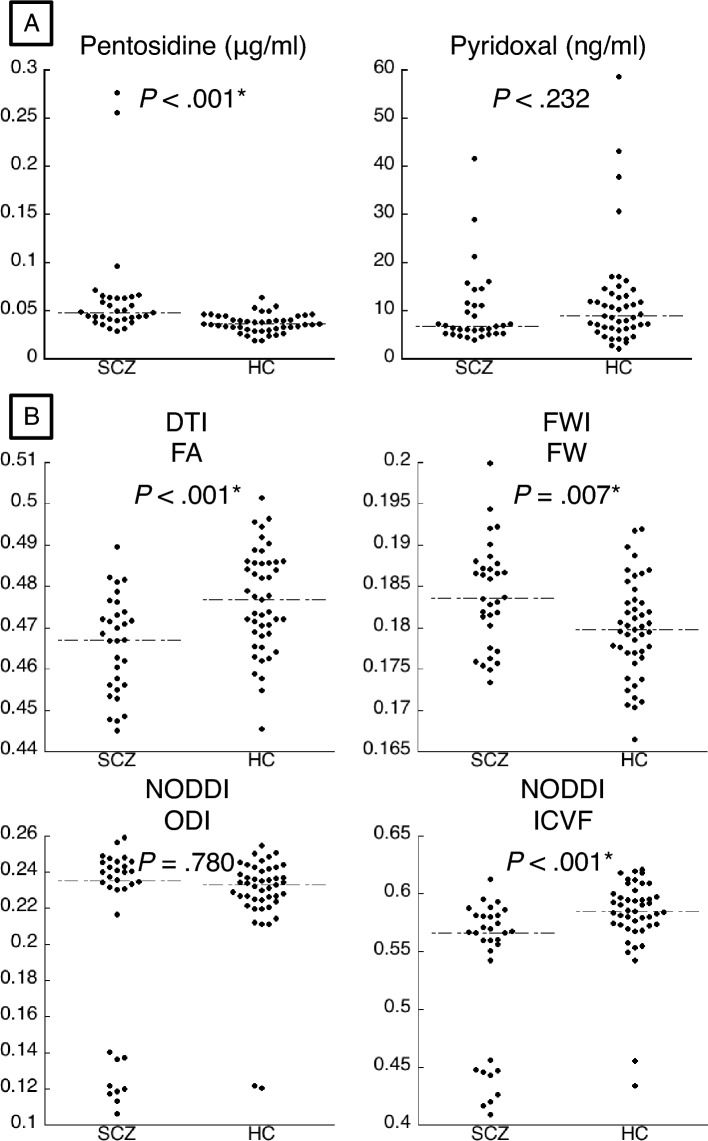
Dot plots of carbonyl stress markers and white matter measures. Dot plots of (**A**) pentosidine and pyridoxal, (**B**) fractional anisotropy (FA) of Diffusion Tensor Imaging (DTI), free water (FW) of free water imaging (FWI), and orientation dispersion index (ODI), intra-cellular volume fraction (ICVF) of Neurite Orientation Dispersion and Density Imaging (NODDI) in the schizophrenia (SCZ) and healthy control (HC) groups are indicated. Median values of A) plasma pentosidine level (SCZ: 0.049 µg/ml, HC: 0.037 µg/ml,), serum pyridoxal level (SCZ: 6.900 ng/ml, HC: 9.000 ng/ml), (**B**) FA (SCZ: 0.467, HC: 0.477), FW (SCZ: 0.184, HC: 0.180), ODI (SCZ: 0.235, HC: 0.233), ICVF (SCZ: 0.566, HC: 0.585) are indicated by lines.

We compared plasma pentosidine and serum pyridoxal between groups, with a significance level of *P* < 0.025 (= 0.05/2 carbonyl stress markers). Compared with the healthy (HC) group, the schizophrenia (SCZ) group had a significantly higher plasma pentosidine level, while there was no significant difference in the serum pyridoxal level.

### Group differences in white matter measures (Fig. 1B)

We also compared mean values of FA, free-water index of FWI (FW), and orientation dispersion index (ODI) and intracellular (intra-neurite) volume fraction (ICVF) of NODDI between groups, with a significance level of *P* < 0.0125 (= 0.05/4 white matter measures). Mean FA and ICVF were significantly decreased (FA: r (effect size of Mann–Whitney U) = − 0.398, *P* < 0.001, ICVF: r = − 0.439, *P* < 0.001), whereas those of free FW were increased (r = − 0.306, *P* = 0.007) in SCZ. We found no group differences in ODI (r = − 0.032, *P* = 0.780).

### Correlation between carbonyl stress markers and white matter measures (Fig. 2)

**Figure 2:**
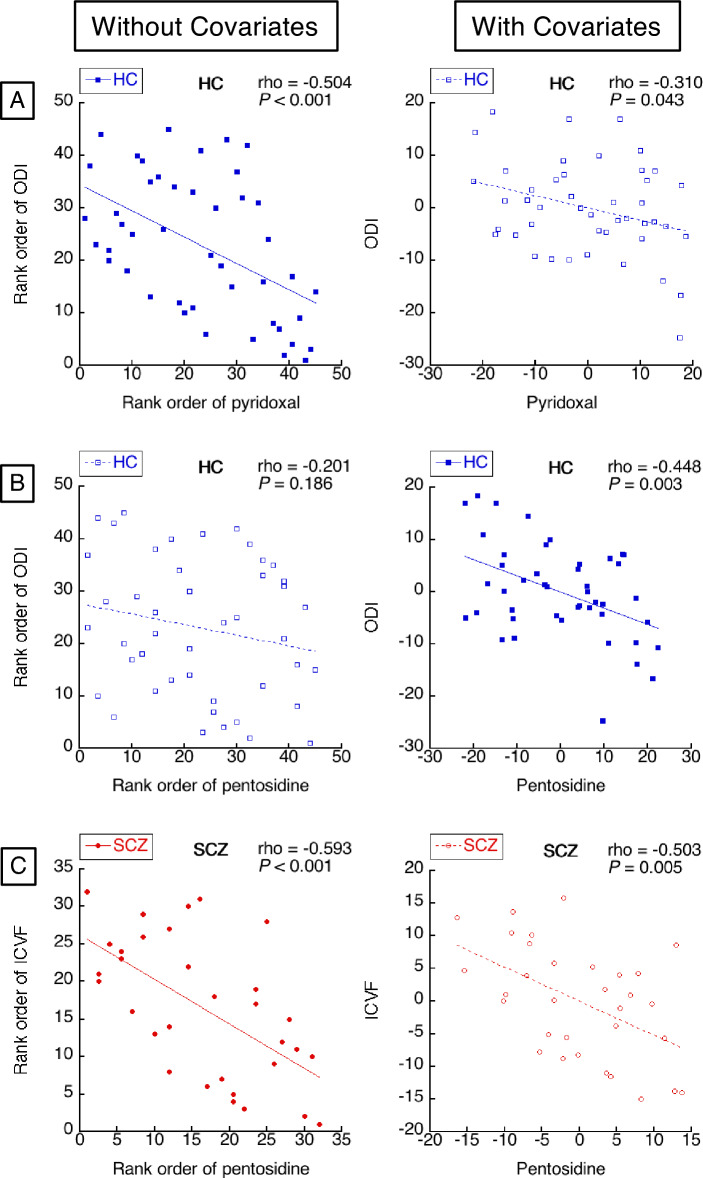

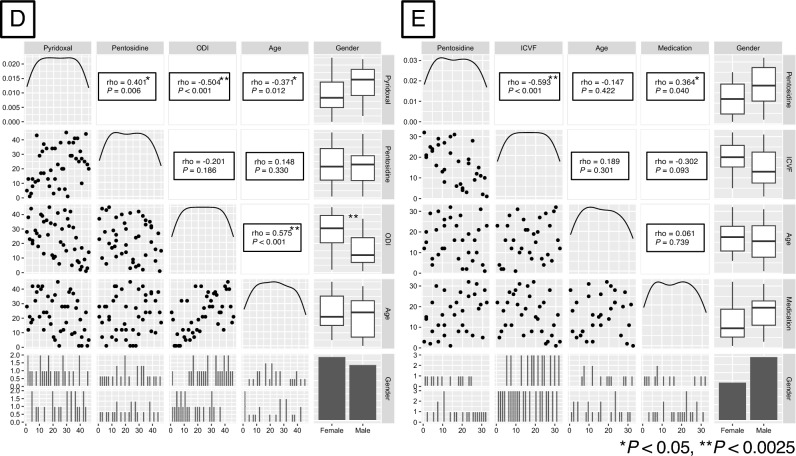
((**A**)-left) The healthy controls (HC) showed significant negative correlation between serum pyridoxal and orientation dispersion index (ODI), but in (A)-right) the result disappeared after controlling for age and gender. ((**B**)-left) LHC did not show significant correlation between plasma pentosidine and ODI, but in (B)-right) the negative correlation disappeared after controlling for age and gender. (C)-left) The schizophrenia (SCZ) group showed a significant negative correlation between plasma pentosidine and intra-cellular volume fraction (ICVF), but in (**C**)-right) the result disappeared after controlling for age, gender, and medication. Red circle markers (●) represent SCZ subjects and blue square markers (■) represent HC subjects. To aid visualization, Spearman's rank orders are shown on both the X and Y axes. The regression line is shown in each graph. Significant results are indicated by filled markers (●, ■) and solid regression lines (—, —), and non-significant results are indicated by open markers (○, □) and broken lines (---, ---). (**D**) The scatter plot matrix of HC. Other than the significant correlations between serum pyridoxal and ODI shown above, we found significant negative correlations between age and ODI and significant group difference of ODI between male and female. (**E**) The scatter plot matrix of SCZ. No other significant correlations were found than between plasma pentosidine and ICVF as shown above.

To assess the association between carbonyl stress and white matter, Spearman’s simple rank correlation analyses between carbonyl stress markers (pentosidine and pyridoxal) and white matter measures (FA, FW, ODI, and ICVF) were performed in each of the groups. The significance level was *P* < 0.0031 (= 0.05 divided by 2 groups, 2 carbonyl stress markers, and 4 white matter measures).

Because age, gender, and antipsychotic medication are known to affect carbonyl stress markers^15,24–31^ and white matter measures^32–36^, we also performed Spearman’s partial correlation analyses using these as control variables, with the same significance level (*P* < 0.0031).

We found a significant negative correlation between serum pyridoxal and ODI in HC without controlling for age and gender (Spearman’s ρ =  − 0.504, *P* < 0.001, Fig. 2A-left), which became non-significant at a corrected level after controlling for these variables (Spearman’s ρ = − 0.310, *P* = 0.043, Fig. 2A-right). While we did not find significant correlation between plasma pentosidine and ODI in HC by the simple correlation analysis (ρ = − 0.201, *P* = 0.186, Fig. 2B-left), we did find a significant negative correlation between them after controlling for age and gender (ρ = − 0.448, *P* = 0.003, Fig. 2B-right). A significant negative correlation between plasma pentosidine and ICVF in SCZ was found without controlling age, gender, and medication (ρ = − 0.593, P < 0.001, Fig. 2C-left), which turned into a trend level after their control (ρ = − 0.503, *P* = 0.005, Fig. 2C-right). No other significant correlations were found.

To clarify the effects of age, gender, and medication, we exhaustively performed simple Spearman’s rank correlations between serum pyridoxal, plasma pentosidine, ODI, age, and gender in HC (Fig. 2D), and between plasma pentosidine, ICVF, age, gender, and medication in SCZ (Fig. 2E). The significance level was set at *P* < 0.0025 (= 0.05 divided by _5_C_2_ and 2 groups). Other than the above-reported significant simple correlations between serum pyridoxal and ODI in HC and between plasma pentosidine and ICVF in SCZ, we found a significant negative correlation between age and ODI (ρ = 0.575, *P* < 0.001), and a significant group difference of ODI between male and female (male < female, r = − 0.512, *P* < 0.001) in HC. We did not find any other significant correlations in SCZ.

To clarify the possible interaction effects of carbonyl stress markers and the control variables of age, gender, and antipsychotic medication (chlorpromazine equivalent) on white matter alteration, we also performed Spearman’s partial correlation analyses between white matter measures and each of interaction terms. We did not find any significant interaction in both groups.

## Discussion

The main findings of this study are: (1) Schizophrenia patients showed decreased neurite density (that is, apparent axonal density in white matter) and increased CSF/edema; (2) Enhanced carbonyl stress was related to decreased apparent axonal density in SCZ; (3) Carbonyl stress indexed by plasma pentosidine and serum pyridoxal was associated with axonal microstructure in HC and SCZ, the significance levels of which changed with or without the effects of age, gender, and medication. The details will be discussed below.

Our DTI result of decreased FA in SCZ supports previous robust findings using DTI^7–12^. Our FWI results of increased FW are consistent with a previous study^37^ but inconsistent with studies of no increase of FW^38–40^ in SCZ. Our NODDI findings of decreased ICVF are in agreement with a previous study^41^. Our finding of no change of ODI is inconsistent with a study revealing increased ODI in SCZ^38^, although that study used single-shell diffusion data. In our study, we used two-shell data acquisition and a preprocessing pipeline^42^ based on the original method^23^, which assures the quality of raw data and preprocessing. In addition to the above, our previous studies using microtomography showed that neurite curvature was negatively correlated with its diameter^43^ and positively correlated with the capillary curvature^44^ in SCZ. Taken together, we can at least understand that decreased ICVF, which can be due to curved neurites along with curved capillaries, contributes to white matter abnormality in SCZ. This might be accompanied by increased CSF/edema, indicating neuroinflammatory change as was previously shown in carbonyl stress model mouse^45^.

By simple correlation analyses, we found that increased pyridoxal was negatively associated with decreased ODI (axonal orientation dispersion) in HC. After controlling for age and gender, this apparent negative association disappeared and a direct negative association between pentosidine and ODI appeared in HC. Age was reported to relate to ODI increase, which was to be exponential after the fourth decade^33^. Most of the subjects in this study were older than the fourth decade (SCZ: N = 21, HC: N = 25). Furthermore, our data showed a significant group difference of ODI between males and females (male < female). Taken together, we can consider that the associations between age and ODI, and between gender and ODI related the apparent association between serum pyridoxal and ODI in HC. On the other hand, there was a direct negative association between pentosidine and ODI in HC, while age and gender inversely related the positive relationship between them.

We found an association between increased pentosidine and decreased ICVF (apparent axonal density in white matter) only in SCZ for simple correlation analysis, which turned into trend-level after controlling for the effects of age, gender, and medication. Since both plasma pentosidine and ICVF had no significant relationships with age, gender, and medication, this reduction of significance may be caused by the reduced degree of freedom from 30 to 27. Another possibility is that individual weak associations as revealed in previous studies^2–5,15,24,25^ might have a relating effect in the inverse direction when combined. Previous in-vitro studies showed that enhanced carbonyl stress was related to shortened neurites^16^ as well as decreased myelination^19^. A postmortem study also revealed a relationship with curved neurites^18^, the association of which may be mediated by a microinflammatory process^46^. Our current finding of apparent axonal density reduction as well as increased CSF/edema is consistent with this concept. Also, we found that the patient with the highest plasma pentosidine level had the lowest level of ICVF. This may imply that there exists a common pathology among the high-carbonyl-stress subgroup and the altered-apparent-axonal-density subgroup. Further studies using drug-naïve and/or at-risk mental state populations would broaden the current findings.

This study has several limitations that need to be considered. First, we have to remember that we cannot completely eliminate the effects of medications by modeling them as covariates alone. Further studies with drug-naïve or drug-free patients would be required. In addition, factors such as exercise, dietary habits, and smoking were not measured or controlled in the current study, and they could affect carbonyl stress and MRI measures. Second, we used peripheral carbonyl stress markers in this study, but their relationship to carbonyl stress in the central nervous system is unclear. Further studies need to elucidate the relationship between peripheral and central carbonyl stress. Third, this study had a cross-sectional design. Investigation of longitudinal changes of carbonyl stress and white matter measures could help to elucidate the causal relationship between these two variables. Fourth, the b-values used for calculating DTI in the current method are slightly lower than the commonly used values (e.g. b = 1000 s/mm^2^). This may have biased the current results, and thus they may need further investigation in terms of the optimality of the b-value for DTI in the future, since recent studies have suggested that DTI with higher b-values of (e.g. 3000 s/mm^2^) is more sensitive to the neurite changes^47^.

Using multiple MRI modalities, we found an association between enhanced carbonyl stress and axonal white matter microstructural abnormality in patients with schizophrenia. This study thus provides insights into the carbonyl stress-based white matter pathophysiology of schizophrenia and could contribute to the development of novel treatments from the viewpoint of carbonyl stress.

## Methods

### Participants

Thirty-two patients with schizophrenia (SCZ: 20 men and 12 women, median [IQR] age = 43.00 [21] years) were recruited. Each patient satisfied the criteria for schizophrenia based on the Structural Clinical Interview for DSM-IV Axis I Disorders (SCID) Patients Edition, Version 2.0. None of the patients were comorbid with other psychiatric disorders. Their psychopathology was assessed using the Positive and Negative Syndrome Scale^48^. All patients were receiving antipsychotic medication at the time of the study (typical [n = 3], atypical [n = 25], typical and atypical [n = 4]). Chlorpromazine equivalents were calculated according to the Practice Guideline for the Treatment of Schizophrenia Patients^49,50^.

Forty-five healthy individuals (HC: 21 men and 24 women, age = 40.00 [11] years) were recruited as control group. Participants were evaluated using the SCID Non-patient Edition, Version 2.0. They had no history of psychiatric disorders and no first-degree relatives who had experienced psychotic episodes. Exclusion criteria for both groups were as follows: a history of head trauma, neurological disease, severe medical disease that could affect brain function, or substance abuse. Participants with high creatinine (> 1.04 mg/dl), high glycohemoglobin A_1C_ (> 6.5%), or who were currently medicated with a vitamin were also excluded so as not to affect the blood sample measurements of pentosidine and pyridoxal. After receiving a complete description of the study, written informed consent was obtained from all participants. This study was approved by the Committee on Medical Ethics of Kyoto University and was conducted in accordance with the Code of Ethics of the World Medical Association.

### Measurement and group comparisons of pentosidine and vitamin B6

Plasma pentosidine and serum pyridoxal were used as markers of carbonyl stress. Fresh plasma and serum samples were obtained from all participants on the same day as MRI scanning. Pentosidine levels were determined using high-performance liquid chromatography as described previously^51^ (see Supplementary Materials). Other parameters (glycohemoglobin A_1C_ and creatinine) were measured from blood samples. The glomerular filtration rate was estimated using the abbreviated Modification of Diet in Renal Diseases study equation^52^. Because two forms of vitamin B6 (pyridoxine and pyridoxamine) were below detectable levels, we used pyridoxal as a vitamin B6 marker.

### MRI scan

All images, which were independent from our previous dataset^15^, were acquired using a 3-Tesla (3 T) MRI unit (MAGNETOM Prisma; Siemens, Erlangen, Germany). T1-weighted images (T1WIs) were acquired using a 3-dimensional (3D) magnetization-prepared rapid gradient echo (3D-MPRAGE) sequence. T2-weighted images (T2WIs) were acquired with 3D T2 Sampling Perfection with Application-optimized Contrasts using different flip angle Evolutions (3D T2-SPACE) sequence. Diffusion-weighted images were acquired using a single-shot spin-echo echo-planar imaging (EPI) sequence with 2 opposing phase encoding directions: from anterior to posterior (AP) and from posterior to anterior (PA). Two b-values of diffusion-weighted data (700 and 2000s/mm^2^), which were optimized for Neurite Orientation Dispersion and Density Imaging (NODDI)^23^, were obtained (see Supplementary Materials). The b = 700 images were used for the following diffusion data preprocessing, and both b = 700 and b = 2000 images were used for NODDI.

### Diffusion data preprocessing

All diffusion data were preprocessed using the HCP pipeline^53^. In short, b0 images were averaged and b0 pairs with two opposite phase encoding directions were used to estimate the B0 inhomogeneity distortion using Topup^54^ implemented in a set of programs of FMRIB Software Library version 5.0.9 (FSL; Oxford Centre for Functional MRI of the Brain, Oxford, UK; www.fmrib.ox.ac.uk/fsl). The eddy-current-induced field inhomogeneities, and the head motion for each image volume, were corrected using FSL's Eddy^55^. Gradient nonlinearity correction was conducted for the diffusion data to remove spatial distortion due to the nonlinearity inherent in the MRI gradient system^56^. The corrected diffusion data were each spatially realigned to the T1WIs using a rigid body transformation with the FSL’s FLIRT boundary-based registration (BBR)^57,58^ followed by FreeSurfer's BBR^59^. Then, a fractional anisotropy (FA) map was obtained by fitting a diffusion tensor model to the data using the DTIFIT program of FSL.

We calculated the temporal signal-to-noise ratio (tSNR) for each voxel by mean/standard deviation of the b0 volumes, which represent a practical imaging quality index^60^. The mean tSNR for the whole brain did not significantly differ between the groups (Table 1).

### Free water imaging

**Figure 3:**
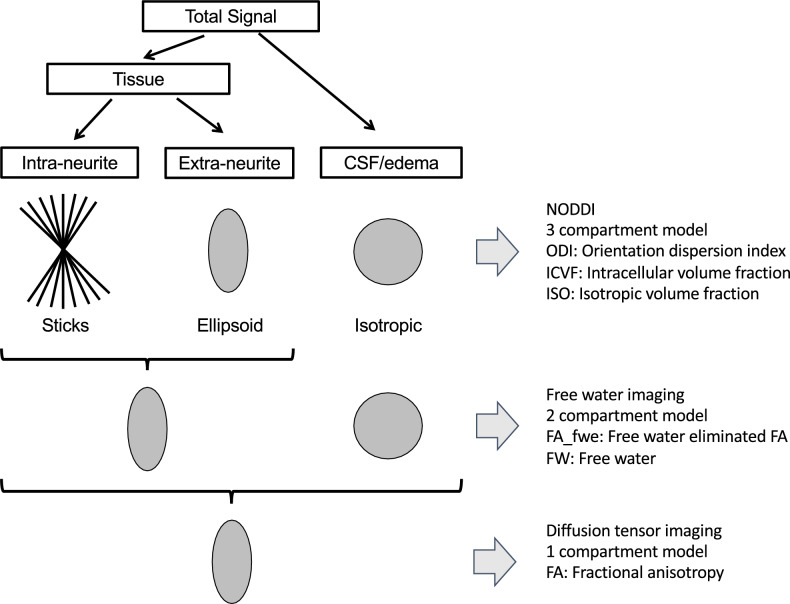
Schemas of diffusion weighted imaging preprocessing. Total diffusion weighted signals are divided into (1) 3 compartments (Neurite Orientation Dispersion and Density Imaging, NODDI), (2) 2 compartments (free water imaging) and 1 compartment (diffusion tensor imaging). Parameters of each preprocessing model are shown on the right part of this figure. This figure is modified from one on the website (http://mig.cs.ucl.ac.uk/index.php?n=Tutorial.NODDImatlab).

**Figure 4:**
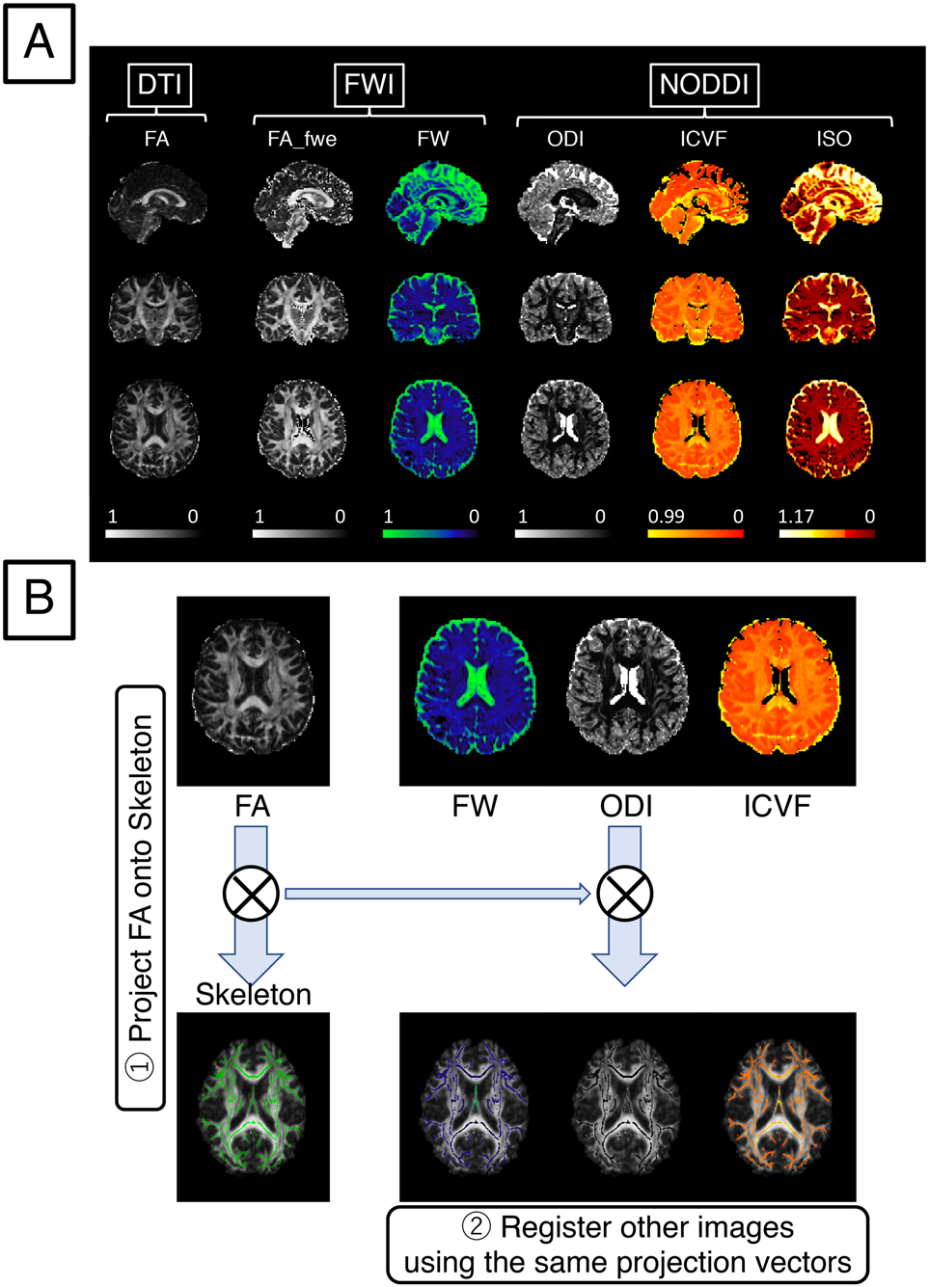
Maps of white matter measures and analysis pipeline of this study. (**A**) Examples of Fractional Anisotropy (FA) of Diffusion Tensor Imaging (DTI); free water eliminated fractional anisotropy (FA_fwe) and free water (FW) of free water imaging (FWI); orientation dispersion index (ODI), intra-cellular volume fraction (ICVF), and isotropic volume fraction (ISO) of Neurite Orientation Dispersion and Density Imaging (NODDI). A color bar of the signal intensity is indicated below each map. (**B**) All steps to project each map onto the skeleton mask are as follows: (1) create FA, create the skeleton mask of FA, and project local maxima of FA, (2) project FW, ODI, and ICVF onto the skeleton mask using the same projection vectors as used in step 1.

Free water imaging^21,61^ models water diffusion in each voxel by a 2-compartment model of tissue (intra- and extra-neurite) and CSF/edema spaces. Increase of the latter is considered to be a surrogate marker of edematous (inflammatory) change^22^ (Fig. 3). This technique generates free-water-eliminated FA (FA_fwe, intracellular water space, Figuress 1, 2A) maps that are more sensitive to microstructural change than a single tensor FA image^21^, and also free water images (FW, extracellular water space, Figs. 3, 4A)^62^ (see Supplementary Materials).

### Neurite orientation dispersion and density imaging (NODDI)

NODDI^23^ models white matter brain microstructure in each voxel by 3 compartments. The intra-neurite compartment (neurite = axon in white matter and axon/dendrite in gray matter) is modeled as restricted diffusion by membrane or myelin; the extra-neurite compartment (outside of neurites $\approx$ glial cells) is modeled as anisotropic hindered diffusion; the CSF/edema compartment is modeled as isotropic diffusion (Fig. 3). This technique generates a volume fraction of the intracellular compartment (intracellular volume fraction, ICVF, Fig. 4A), a volume fraction of the CSF/edema compartment (isotropic volume fraction, ISO, Fig. 4A), and the orientation dispersion index (ODI, Figs. 3, 4A) (see Supplementary Materials).

### Tract-based spatial statistics (TBSS)

After visual inspection of all the preprocessed MRI data, we used Tract-Based Spatial Statistics (TBSS, version 1.2 of FSL)^63^ (see Supplementary Materials) to calculate mean white matter values of the above indices. The voxel values of each subject’s normalized FA map were projected onto the mean “skeleton” (Fig. 4B) by identifying the local maxima along the perpendicular direction from the skeleton. Each subject’s maps of FW for inflammatory change, and ODI and ICVF for neurite qualitative and quantitative evaluation were also projected onto the skeleton using the same projection vectors (Fig. 4B). Mean values of the skeletonized data were calculated within the skeleton mask, and they were used for the group comparisons and correlational analyses as described in the following section.

### Statistical analyses

Because all the carbonyl stress and imaging indices above were not normally distributed in both the HC and SCZ groups, non-parametric analyses by Mann–Whitney U tests for group comparisons and Spearman’s correlation coefficient for correlational analyses were used. All analyses were performed using SPSS version 23 (SPSS Inc., Chicago, IL, USA). The significance levels were set according to Bonferroni correction.

We compared plasma pentosidine and serum pyridoxal between groups, with a significance level of *P* < 0.025 (= 0.05/2 carbonyl stress markers).

We also compared mean FA, FW, ODI, and ICVF values between groups, with a significance level of *P* < 0.0125 (= 0.05/4 white matter measures).

Spearman’s simple and partial correlation analyses between carbonyl stress and white matter measures were performed in each of the groups, with or without control variables of age, gender, and antipsychotic medication (chlorpromazine equivalent)^49,50^. The significance level was *P* < 0.0031 (= 0.05 divided by 2 groups, 2 carbonyl stress markers, and 4 white matter measures).

### Effects of age, gender, and medication

To examine the effects of age, gender, and medication on the association between carbonyl stress and white matter alteration, we performed simple Spearman’s correlation analyses between serum pyridoxal, plasma pentosidine, ODI, age, and gender in HC, and between plasma pentosidine, ICVF, age, gender, and medication in SCZ. The significance level was set at *P* < 0.0025 (= 0.05 divided by _5_C_2_ and 2 groups).

### Interaction effects of age, gender, and medication

To clarify the possible interaction effects of age, gender, and antipsychotic medication (chlorpromazine equivalent) on the relationship between carbonyl stress markers and white matter alteration (ODI in HC and ICVF in SCZ, respectively), we also performed Spearman’s partial correlation analyses for an interaction term while controlling for other terms. The interaction terms were computed as the dot-product of the 2 carbonyl stress markers (plasma pentosidine or serum pyridoxal) and the control variables of age or gender for HC, and the carbonyl stress marker (plasma pentosidine) and the control variables of age, gender, or medication for SCZ, respectively. All variables were mean-centered before computing the interactions. The significance level was set at P < 0.0125 (= 0.05 divided by (4 interactions between 2 carbonyl stress markers, and age/gender) in HC, and P < 0.0167 (= 0.05 divided by (3 interactions between plasma pentosidine and age/gender/medication) in SCZ.

## Supplementary Information

﻿Supplementary Information.

## Data Availability

The data analyzed in this study are available from the corresponding author on reasonable request.
