# Supplementary material for: Association between enhanced carbonyl stress and decreased apparent axonal density in schizophrenia by multimodal white matter imaging
Source: Sci Rep. 2023 Jul 27;13:12220. doi: 10.1038/s41598-023-39379-w (PMC10374594; doi:10.1038/s41598-023-39379-w)
Supplement: Supplementary file 1 — ﻿Supplementary Information. [file 41598_2023_39379_MOESM1_ESM.docx]

**Supplementary Materials**

Measurement and group comparisons of pentosidine and vitamin B6

Plasma samples were lyophilized and hydrolyzed in 100 µl of 6N hydrochloric acid at 110°C under nitrogen for 16 h. Samples were then neutralized with 100 µl of 5N sodium hydroxide and 200 µl of 0.5 M sodium phosphate buffer (pH 7.4), filtered through a 0.5-µm filter, and finally diluted with phosphate-buffered saline. A sample containing 25 µg of protein was injected into a high-performance liquid chromatography system and fractionated on a C18 reverse-phase column. Effluent was monitored at excitation-emission wavelengths of 335/385 nm using a fluorescence detector (RF-10A; Shimadzu, Kyoto, Japan). Synthetic pentosidine was used to obtain a standard curve. Serum levels of the three forms of vitamin B6 (pyridoxine, pyridoxal, and pyridoxamine) were determined using high-performance liquid chromatography at a private clinical laboratory test company (SRL, Tokyo, Japan) according to a previously described method ^1^.

MRI scan

All images were acquired using a 3-Tesla (3T) MRI unit (MAGNETOM Prisma; Siemens, Erlangen, Germany. Maximum gradient amplitude: 80 mT/m; slew rate: 200 T/m/s) and a receiver-only 64-channel phased-array head and neck coil. T1-weighted images (T1WIs) were acquired in sagittal orientation using a 3-dimensional (3D) magnetization-prepared rapid gradient echo (3D-MPRAGE) sequence (repetition time (TR), 2000 ms; echo time (TE), 2.13 ms; inversion time, 952 ms; field of view (FOV), 230×230 mm; matrix, 256×256; resolution, 0.9×0.9×0.9 mm^3^; sections, 208; parallel imaging factor). T2-weighted images (T2WIs) were acquired with 3D T2 Sampling Perfection with Application-optimized Contrasts using different flip angle Evolutions (3D T2-SPACE) sequence (TR, 3000 ms; TE, 538 ms; the other parameters were the same as for 3D-MPRAGE). Diffusion-weighted images were acquired in slice orientation parallel to the anterior and posterior commissures using a single-shot spin-echo echo-planar imaging (EPI) sequence (a monopolar Stejskal-Tanner type sequence) with multiband capability ^2,3^ using fixed parameters (TR, 3500 ms; TE, 75.8 ms; FOV, 200×200 mm; matrix, 118×118; resolution, 1.7×1.7×1.7 mm^3^; slices 86; multiband factor) with 2 opposing phase encoding directions: from anterior to posterior (AP) and from posterior to anterior (PA). Six without diffusion weighting (B0 images), 16 b=700 s/mm^2^, and 32 b=2000 s/mm^2^ diffusion-weighted data were scanned in the AP direction and 7 b0, 16 b=700, and 32 b=2000 data were scanned in the PA direction. There were 109 volumes in total.

Free water imaging

Free water imaging ^4,5^ models water diffusion in each voxel by a 2-compartment model of intra- and extra-cellular water space. The normalized signal of diffusion MRI ($A$) is written as follows:

$$A=C_{tissue}+C_{water}=\int A_{tissue}\left( D \right)+\left( 1-f \right)A_{water}$$

where $C$ is a cellular compartment, $D$ is diffusion tensor, and $f$ is a fractional volume of a tissue compartment. Multi-shell diffusion weighted images were fitted to a regularized bi-tensor model to generate free-water-eliminated FA (FA_fwe, intracellular water space, Figures 3, 4A) maps and free water images (FW, extracellular water space, Figures 3, 4A) ^6^.

Neurite Orientation Dispersion and Density Imaging (NODDI)

NODDI ^7^ models white matter brain microstructure in each voxel by 3 compartments. The intracellular compartment (neurite = axon in white matter and axon/dendrite in gray matter) is modeled as restricted diffusion by membrane or myelin; the extracellular compartment (outside of neurites $\approx$ glial cells) is modeled as anisotropic hindered diffusion); the CSF/edema compartment is modeled as isotropic diffusion (Figure 3). Importantly, in white matter the intracellular compartment indicates axon. The normalized signal of diffusion MRI ($A$) is written as follows:

$A=\left( 1-v_{iso} \right)\left( v_{ic}A_{ic}+\left( 1-v_{ic} \right)A_{ec} \right)+v_{iso}A_{iso}$,

where $A_{ic}$ and $v_{ic}$ are the normalized signal and volume fraction of the intracellular compartment (intracellular volume fraction, ICVF, Figure 4A); $A_{ec}$ is the normalized signal of the extracellular compartment; $A_{iso}$ and $v_{iso}$ are the normalized signal and volume fraction of the CSF/edema compartment (isotropic volume fraction, ISO, Figure 4A). In the intracellular compartment, we also obtain the orientation dispersion index (ODI, Figures 3, 4A) using Watson distribution, which represents neurite orientation dispersion ^7^. The calculation was conducted using Accelerated Microstructure Imaging via Convex Optimization AMICO ^8^ implemented in MATLAB (http://www.mathworks.com/).

Tract-Based Spatial Statistics (TBSS)

To calculate mean white matter values of the above indices, we used Tract-Based Spatial Statistics (TBSS, version 1.2 of FSL) ^9^. In brief, all FA data were spatially normalized, averaged to create a mean FA image, and then thinned to create an original mean FA “skeleton”, taking only the centers of white matter tracts common to all subjects. This original mean FA skeleton was thresholded at a value of 0.2 to create a mean FA skeleton mask (Figure 4B). The voxel values of each subject’s normalized FA map were projected onto the mean skeleton by identifying the local maxima along the perpendicular direction from the skeleton. Each subject’s FW, ODI, and ICVF were also projected onto the skeleton using the same projection vectors (Figure 4B).

**References**

1 Bisp, M. R., Bor, M. V., Heinsvig, E. M., Kall, M. A. & Nexo, E. Determination of vitamin B6 vitamers and pyridoxic acid in plasma: development and evaluation of a high-performance liquid chromatographic assay. Anal. Biochem. 305, 82-89, https://doi.org/10.1006/abio.2002.5638 (2002).

2 Moeller, S. et al. Multiband multislice GE-EPI at 7 tesla, with 16-fold acceleration using partial parallel imaging with application to high spatial and temporal whole-brain fMRI. Magn Reson Med 63, 1144-1153, https://doi.org/10.1002/mrm.22361 (2010).

3 Feinberg, D. A. et al. Multiplexed echo planar imaging for sub-second whole brain FMRI and fast diffusion imaging. PLoS One 5, e15710, https://doi.org/10.1371/journal.pone.0015710 (2010).

4 Pasternak, O., Sochen, N., Gur, Y., Intrator, N. & Assaf, Y. Free water elimination and mapping from diffusion MRI. Magn Reson Med 62, 717-730, https://doi.org/10.1002/mrm.22055 (2009).

5 Pasternak, O., Sochen, N. & Basser, P. J. The effect of metric selection on the analysis of diffusion tensor MRI data. Neuroimage 49, 2190-2204, https://doi.org/10.1016/j.neuroimage.2009.10.071 (2010).

6 Hoy, A. R., Koay, C. G., Kecskemeti, S. R. & Alexander, A. L. Optimization of a free water elimination two-compartment model for diffusion tensor imaging. Neuroimage 103, 323-333, https://doi.org/10.1016/j.neuroimage.2014.09.053 (2014).

7 Zhang, H., Schneider, T., Wheeler-Kingshott, C. A. & Alexander, D. C. NODDI: practical in vivo neurite orientation dispersion and density imaging of the human brain. Neuroimage 61, 1000-1016, https://doi.org/10.1016/j.neuroimage.2012.03.072 (2012).

8 Daducci, A. et al. Accelerated Microstructure Imaging via Convex Optimization (AMICO) from diffusion MRI data. Neuroimage 105, 32-44, https://doi.org/10.1016/j.neuroimage.2014.10.026 (2015).

9 Smith, S. M. et al. Tract-based spatial statistics: voxelwise analysis of multi-subject diffusion data. Neuroimage 31, 1487-1505, https://doi.org/10.1016/j.neuroimage.2006.02.024 (2006).
